# Supplementary material for: Shifts in attention drive context-dependent subspace encoding in anterior cingulate cortex in mice during decision making
Source: Nat Commun. 2024 Jul 2;15:5559. doi: 10.1038/s41467-024-49845-2 (PMC11220070; doi:10.1038/s41467-024-49845-2)
Supplement: Supplementary file 1 — Supplementary Information [file 41467_2024_49845_MOESM1_ESM.pdf]

# Shifts in attention drive context-dependent subspace encoding in anterior cingulate cortex during decision making

## Supplemental Information

Márton Albert Hajnal[1,*], Duy Tran[2,3], Zsombor Szabó[1], Andrea Albert[1], Karen Safaryan[2], Michael Einstein[2], Mauricio Vallejo Martelo[2], Pierre-Olivier Polack[4], Peyman Golshani[2,5,6, †,*], Gergő Orbán[1, †,*]

1, Department of Computational Sciences, HUN-REN Wigner Research Centre for Physics, Budapest, 1121, Hungary
2, Department of Neurology, David Geffen School of Medicine, University of California, Los Angeles, Los Angeles, CA 90095, United States
3, Albert Einstein College of Medicine, New York, NY 10461, United States
4, Center for Molecular and Behavioral Neuroscience, Rutgers University, Newark, NJ 07102, United States
5, Integrative Center for Learning and Memory, Brain Research Institute, University of California, Los Angeles, Los Angeles, CA 90095, United States
6, West Los Angeles VA Medical Center, CA 90073 Los Angeles, United States
†, Equal contributions
*, Corresponding Authors (hajnal.marton@wigner.hun-ren.hu, pgolshani@mednet.ucla.edu, orban.gergo@wigner.hun-ren.hu)

# Supplementary text

**Proposition 1**. Assuming linear neural activity space representation, with fixed weights, modulatory signals must act on subspaces.

*Proof*. To formalize the problem, suppose $X$ is a vectorspace that represents the space of possible neural population activity. The coordinates for $\mathbf{x} \in X$, $x_1,x_2,...,x_N$ are instantaneous firing rates of each of N neurons. Task related variables can be represented in subspaces of $X$. The results below are equally applicable to more than one dimensional stimulus representations, including two dimensional dependent one-hot encoded otherwise one dimensional stimuli, as well as to multiple modulations: In the equations below only the bases need to be expanded, connection matrices changed to block matrices, standard basis vectors changed to the sum of multiple standard basis vectors. For simplicity of notation, here we restrict the proof to two stimulus subspaces, and minimal encodings in one-dimensional linear subspaces. In addition, as per the premise above, we are dealing with agents that already learned the task, so we will not explore dynamically changing weights. Stimulus encoding subspaces can be found by linear decoders projecting with coefficients $\mathbf{e}_V$, $\mathbf{e}_A \in X$ to $V$ and $A$, the subspaces of $X$ where the visual and auditory stimulus related activity resides. We use the format, $\mathbf{e}_{S.}$, as basis for one dimensional subspaces $S \subseteq X$ expressed in the native coordinate system of $X$. Note, that $V$ and $A$ are subspaces, as long as linear independence holds, i.e. while the absolute value of the correlation between visual and auditory related activity < 1, but this is not enough for non-interfering modulations. We focus our assessment on orthogonal subspaces (where correlation is 0, and $\mathbf{e}_V \cdot \mathbf{e}_A = 0$), and expand on linearly independent non-orthogonal subspaces in Corollary 2 (iii). We will also assume stimulus input to the neurons in $X$ are constant, $v$ and $a$, so any further manipulation to stimulus must happen within $X$. In this setup the total stimulus related activity, $\mathbf{s}$, (Fig. 5a) is:

$$\mathbf{s} = \mathbf{v} + \mathbf{a} = v\,\mathbf{e}_V + a\,\mathbf{e}_A \in V + A \subseteq X. \quad (1)$$

The task requires that $X$ has an abstract one dimensional subspace, $D \subseteq X$, which has activity $\mathbf{d} = d\,\mathbf{e}_D \in D$ that represents the final output, i.e. the 'decision' of these neurons. The map to this decision subspace can be performed by a linear operator $\mathrm{F} \in \mathcal{L}(X)$. With the synaptic weights as components of the map, and initial activity $\mathbf{x} \in X$, the transformation is

$$d\,\mathbf{e}_D = \mathrm{F}(\mathbf{x}), \quad (2)$$

or in neural coordinates:

$$d = \mathbf{f}\,\mathbf{x} = \sum_n f_n x_n. \quad (3)$$

At this point we introduce a modulation $u$, that changes $d$. Both the map F, or the activity, $\mathbf{x}$ can depend on the modulation, but the weights $f_n$ are constants by the conditions of the proposition. This leaves us to implement modulation dependent computation within $\mathbf{x}$. We define stimulus-unrelated activity, $\mathbf{z} \in Z$, in the complement of $V + A$, so that $(V + A) \oplus Z =$

$X$; **z** can also have modulation-dependent terms, hence **z**(u). Thus, we need to solve the following problem for a linear modulation map, **m**(u) $\in X$, that maps the modulation, $u$, to $X$:

$$d\,\mathbf{e}_D = F(\,v\,\mathbf{e}_V + a\,\mathbf{e}_A + \mathbf{m}(u) + \mathbf{z}(u)\,). \quad (4)$$

First, it is clear that the dot product $\mathbf{m}\cdot(\mathbf{e}_V+\mathbf{e}_A) \neq 0$, otherwise **m** does not influence stimulus related activity. Second, by definition we have incorporated into **z** any stimulus-unrelated terms from the basis decomposition of **m**(u), including modulation-dependent stimulus-unrelated terms, denoted as **z**(u). These two items can only occur simultanously, if **m**(u) maps only to the subspace spanned by the linear combination of $\mathbf{e}_V$ and $\mathbf{e}_A$., i.e. to $V + A$.

Thus, a modulating signal that aims to influence the decision which in turn depends on stimulus related activity must map onto the stimulus subspace. ■

**Definition**. Context is defined as the set of stimulus the decisions need to be based on. Thus in each context one set is relevant and the others are irrelevant. More specifically in our paradigm the contexts are defined by the relevant stimulus modality.

**Corollary 1**. Context modulation gates activity on the stimulus subspaces by self-enhancement and mutual inhibition.

*Proof*. Let us formalise context modulation. Let the modulation map, **m** from Proposition 1, with range $V + A$ depend on a vector-valued context modulation term, u = **c**. In $X$ coordinates:

$$\mathbf{m}(\mathbf{c}) = c_1\,\mathbf{e}_V + c_2\,\mathbf{e}_A, \quad (5)$$

where $\mathbf{c} = \begin{pmatrix} c_1 \\ c_2 \end{pmatrix}$ is the one-hot encoded context vector that takes values $\begin{pmatrix} 1 \\ 0 \end{pmatrix}$ or $\begin{pmatrix} 0 \\ 1 \end{pmatrix}$ for visual or auditory context, respectively. It is clear that for self-enhancement, the coordinates of c are in the appropriate order, while for inhibition the equation has the form of $\mathbf{m}(\mathbf{c}) = -\,c_2\,\mathbf{e}_V - c_1\,\mathbf{e}_A$. This notation has some complicated case by case description requirements, so we improve on the notation.

We would like to express **m**(**c**) in matrix notation; the benefits of why it is a useful notation will be expanded below. We start with the case for inhibition. Observe that **m**(**c**) must contain a multiplicator, $\mathbf{M} = \begin{pmatrix} 0 & -1 \\ -1 & 0 \end{pmatrix}$, that is anti-diagonal, signifying that the irrelevant subspace is the opposite of the context-relevant subspace corresponding to the changed order and negative sign above, and with negative components so that its effect is inhibition:

$$\mathbf{m}(\mathbf{c}) = \mathbf{M}\,\mathbf{c}\,[\,\mathbf{e}_V\ \mathbf{e}_A\,], \quad (6)$$

Where [ . ] is a matrix from column vectors for the basis vectors of $V + A$. Applying this context dependent modulation as inhibition to the stimulus related input yields the correct order and sign as above in the simple notation:

$$d\,\mathbf{e}_D = F\,(\,(v - c_2)\,\mathbf{e}_V + (a - c_1)\,\mathbf{e}_A\,) + F(\mathbf{z}). \quad (7)$$

Although the decision can be further influenced by F(**z**), it cannot have stimulus-related projection, as $Z$ and $V + A$ are disjoint by definition. Thus, **m**(**c**) contains all that is both allowed and needed for context specific inhibition of irrelevant stimuli.

Inhibition would reduce the activity in the irrelevant subspace, possibly down to 0. This can be achieved with **M** = $\begin{pmatrix} 0 & -\upsilon \\ -\alpha & 0 \end{pmatrix}$, where the fixed matrix weights, $\upsilon$ and $\alpha$ represent the learned optimal suppression of the context-irrelevant visual and audio subspace activity required for successful task execution.

Using this optimal suppression, the above matrix multiplication in **m**(**c**) gives an intuitive implementation in neural circuits: Context, one-hot encoded in vector **c** as defined above, acts as an input to the stimulus subspace, *V* + *A*, through fixed input connections **M**, resulting in context-gated inhibition of the irrelevant subspace. As a concrete example, the effect of this gated inhibition with F = $\begin{pmatrix} 1 & 1 \end{pmatrix}$, the simplest additive projection mapping to *D*, while disregarding **z** for simplicity, expressed in *V* + *A* coordinates, in the visual context, is:

$$\mathrm{d}(\mathbf{c}) = \mathbf{F}(\mathbf{s} + \mathbf{Mc}) = \begin{pmatrix} 1 & 1 \end{pmatrix} \left( \begin{pmatrix} \nu \\ \alpha \end{pmatrix} + \begin{pmatrix} 0 & -\nu \\ -\alpha & 0 \end{pmatrix} \begin{pmatrix} 1 \\ 0 \end{pmatrix} \right) =$$

$$= \begin{pmatrix} 1 & 1 \end{pmatrix} \left( \begin{pmatrix} \nu \\ \alpha \end{pmatrix} + \begin{pmatrix} 0 \\ -\alpha \end{pmatrix} \right) = \begin{pmatrix} 1 & 1 \end{pmatrix} \begin{pmatrix} \nu \\ \alpha - \alpha \end{pmatrix} =$$

$$= \upsilon, \tag{8}$$

with the last equation holding, when the suppression α removes the auditory activity from the auditory subspace, i.e. *a*-$\alpha$ << $\nu$, thus only the visual activity is projected onto the decision subspace.

Note, that we did not assume the exact place in the brain hierarchy where the inhibition should take place, in other words *X* can be arbitrarily large. It is just necessary that it happens before mapping to the final decision space, *D*, by F. The concrete implementation of inhibition has many biological mechanisms, but ultimately all can be transformed into the format in the proof: Reducing outgoing activity of specific neurons that form the coordinates of the basis for that stimulus encoding subspace.

An enhancement of the relevant stimulus instead of, or beside the suppression of the irrelevant stimulus is also plausible. If the modulator input connections **M** is changed to a diagonal matrix with any positive components $\nu$ and $\alpha$: $\begin{pmatrix} \nu & 0 \\ 0 & \alpha \end{pmatrix}$, it will act as context gated positive feedback. Again, $\nu$ and $\alpha$ are learned optimal enhancement multipliers in a fixed input map. With these connection weights the proof holds for selection with an additional condition. The downstream readout threshold (below which input is discarded) must be set between the enhanced and normal stimulus activity levels; this condition is often true as thresholds are typical parts of neural circuitry.

One can combine self-enhancement and mutual inhibition of subspaces with the qualitative form **M** = $\mathbf{M}_e$ + $\mathbf{M}_i$ = $\begin{pmatrix} 1 & -1 \\ -1 & 1 \end{pmatrix}$.

So far context was regarded as an input from outside *V* + *A*, potentially from outside *X*. Let us assume now a mixed representation of stimuli and a locally computed context from e.g. with a context-invariant reward signal as the only input using the current stimuli and the response choice. Here the role of **M** although remains the same, its domain will equal its range: *V* + *A*.

While there are multiple exact mappings possible, the most efficient encoding of context appears to be when **c** equals **v** $\in$ *V*, or **a** $\in$ *A* in the two contexts respectively.

In summary we proved that in linear subspace representations, a simple context driven switch is capable of selecting the relevant modality with inhibition or enhancement, and with fixed weights this computation has to act on the stimulus subspaces. ■

**Corollary 2**. The proof of Corollary 1 can be generalized with regard to F. The F function on *X* can typically have five additional characteristics in neural circuits: i) it can expand out of the stimulus subspace, ii) connections can allow mixed selectivity, iii) can modulate in non-orthogonal stimulus subspaces, iv) the mapping is composed with nonlinear transfer functions, v) brain circuits and RNNs often compute in recurrent sequential activation.

*Proof*. We address these four problems separately.

i) We can simply disregard any component of F that escapes its interesting part of its range, *D*, as it will not influence downstream decision making.

ii) Although many cells typically operate with mixed selectivity, a number of variables can still be encoded simultaneously within a set of neurons without interference: The basis vectors of such mixed encoding subspaces are rotated from the natural neural coordinate system, preserving orthogonal relations between correlated activity directions, i.e. subspaces. The above proof works in these rotated subspaces as well, **F** feedback connections and **D** decision projection can be transformed so that they operate either or both on rotated domain or range spaces. With the additional use of simple change of basis operators, the form of the proof remains the same.

iii) when **v** and **a** are not orthogonal, the modulation **m** will have components that also modulate the relevant subspace to the extent of the angle between the two subspaces. Still, non-orthogonal stimulus subspaces are modulated in a way that the largest modulation is in the intended direction, while to some extent it spills over to the unintended direction. Therefore, fully orthogonal subspaces are of special interest, because they have zero projection onto each other, therefore the modulation is non-interfering with relevant subspaces.

iv) There can be arbitrary nonlinear computations if a σ nonlinearity is applied: $\sigma \circ$ F. However, if σ is smooth and monotonic, which is true for most biological neurons, the shape of the nonlinearity will not affect the local subspace geometry, nor will it affect the additive inhibition or enhancement. A notable exception is negative sign change by $\sigma$, but that can be addressed by flipped connection weights in elements of F if the operating range of σ requires it, as both F and $\sigma$ are fixed. Thus, all statements on subspaces are also applicable in submanifolds with smooth differential structure.

v) Sequential F can be thought of as a composition F ∘ F ∘ F ∘ … or $\mathbf{F}^t$ after time *t* before opening the gates for output. At the required time point, however, the abstraction must confine the meaningful decision related output activity onto *D*. Should F map stimulus related activity outside D + V + A, an extension to ii), the original *V* + *A* subspace will just need to be unified with the subspace of the range of F where *V* + *A* got mapped, and the above proof works with this larger $V^* + A^* \subseteq X$, and $V \subseteq V^*$, $A \subseteq A^*$. A simple consequence of repeated application of F is that F can take over the role of the connections of the input map, M for maintained activity,

for example, when the input **c** is for a single time point, that gets mapped onto $V + A$, and then can be maintained by F with self-enhancement and mutual feedback similar to **M** for the subsequent time points within $V + A$. This essentially allows for maintaining the short input signal even in absence of a clear task, or stimulus, but within the stimulus subspace $V + A$. However, without the initial context input, even with **M**-like F, the bistable system on its own will not decide on the projection and will stay on its unstable point, eventually randomly landing on one of the fixed states. Note however, that in absence of direct context input, i.e. with locally computed context, sequential processing with F similarly assumes mixed representation of a locally computed context and stimuli within the same subspace. In this case **M** maps from within $V + A$ into $V + A$, and thus **M** equals the asymptotic F raised to the number of time steps until the decision typically needs to be made. This incorporates context computation, mapping onto the entirety of stimulus subspaces for modulation and long-term context maintenance into the single recurrent F operator.

Thus, the proof for Corollary 1 also holds on more realistic nonlinear, larger dimensional, sequentially operating neural manifolds. ■

# Supplementary figures

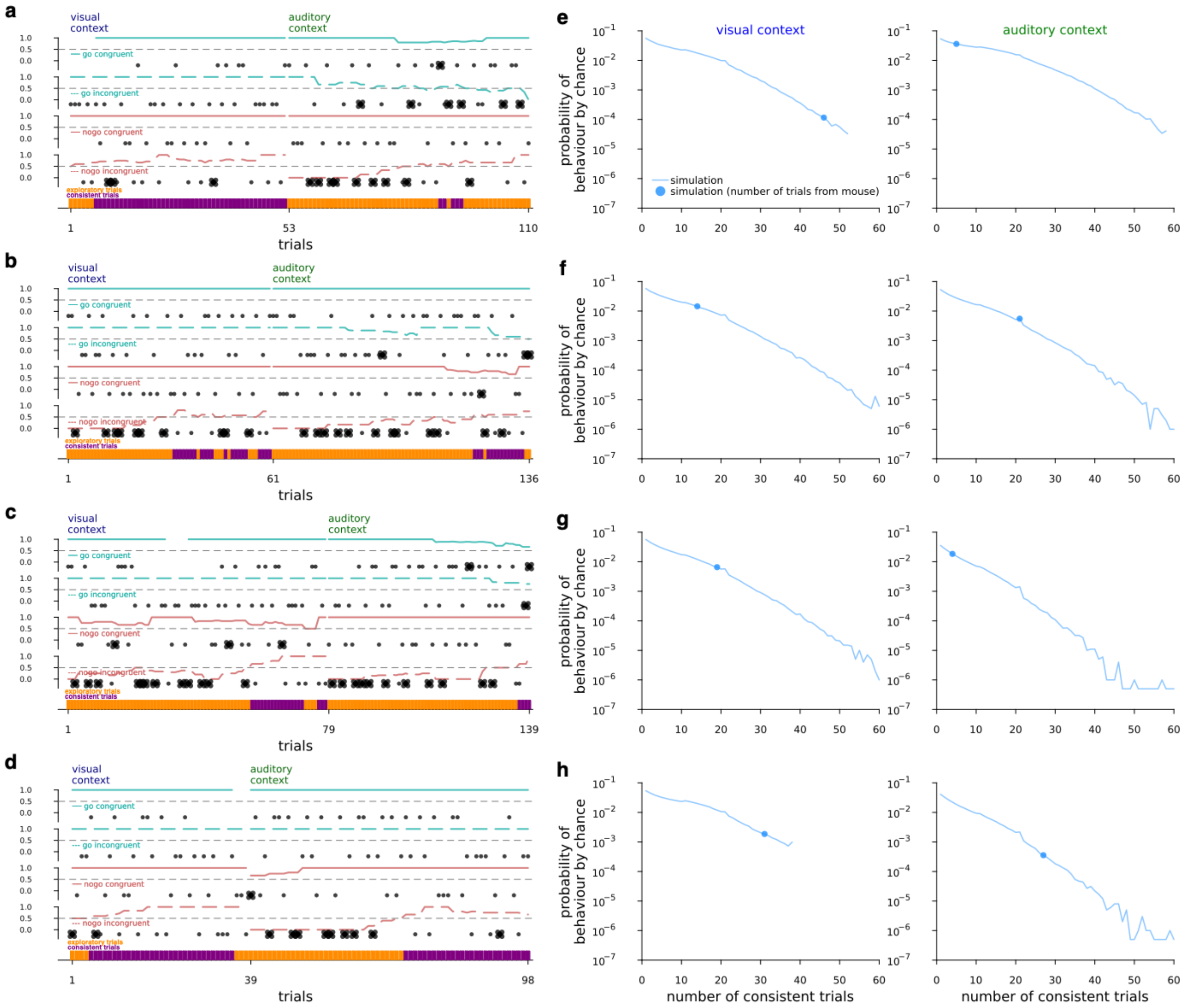


**Supplementary Figure 1**. **Behaviour and probability of performance at chance level**. Rows: n=4 mice with ACC electrode implant. **A-D**, Behaviour of animals for different trial types (*subpanels*). Success and failure in all four trial types are indicated by black filled circles and crosses, respectively. Lines show 21 trial equal-weight moving averages. Trials were defined as 'task-consistent' (*bottom panel, purple*) if the moving average performance of all four trial types were greater than chance, while other parts of the session were termed 'exploratory'. **E-H**, probabilities of the number of consistent trials counted in $10^6$ simulated sessions, where congruent trials were always successful, incongruent trials sampled from the Bernoulli distribution at *p* equalling the empirical lick rate in incongruent trials of the mouse in that context (*blue line*), and the number of consistent trials observed in mice (*blue dots*). Left and right panels show visual and auditory contexts respectively.

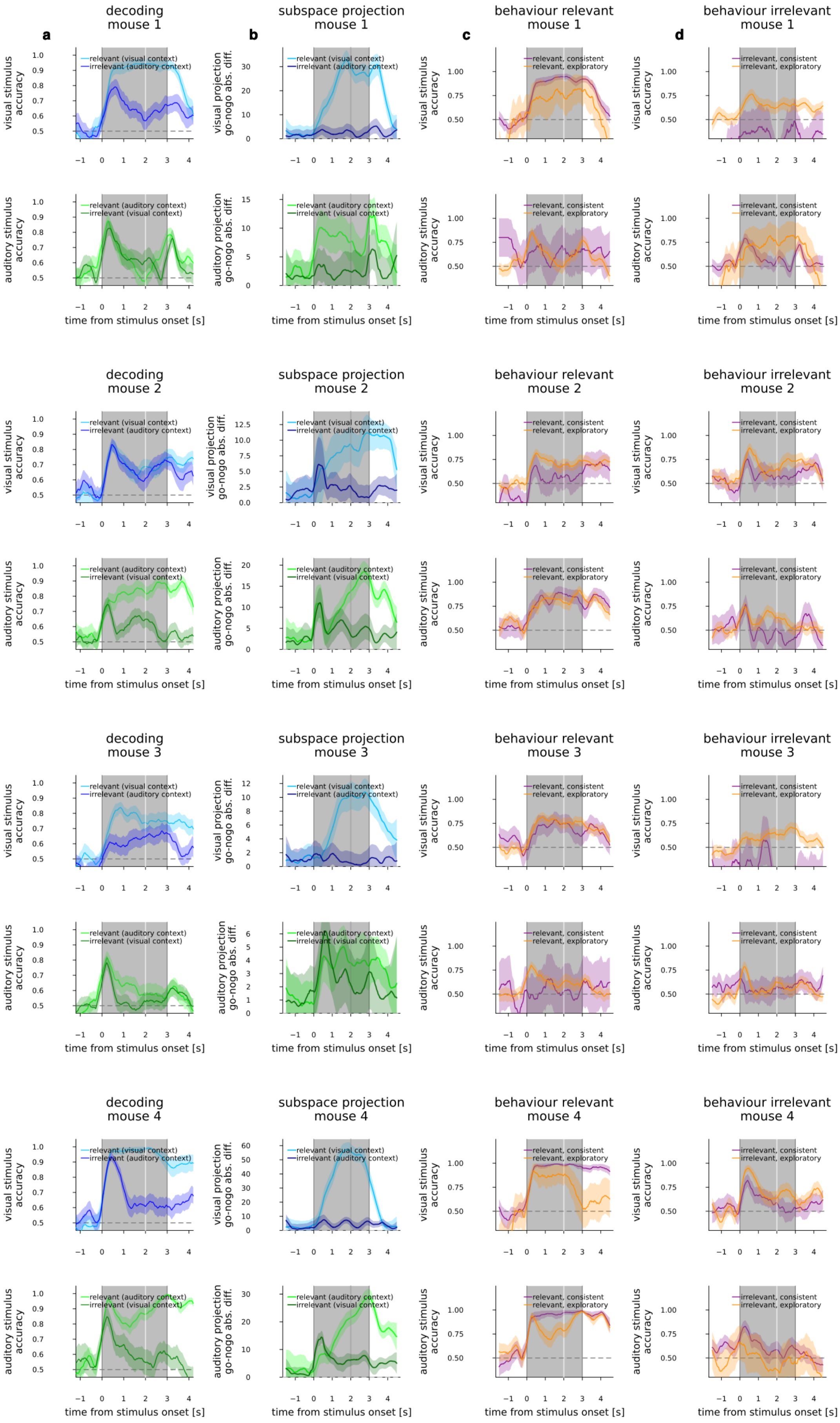

a
b
c
d
decoding
mouse 1
subspace projection
mouse 1
behaviour relevant
mouse 1
behaviour irrelevant
mouse 1
visual stimulus accuracy
auditory stimulus accuracy
visual projection go-nogo abs. diff.
auditory projection go-nogo abs. diff.
relevant (visual context)
irrelevant (auditory context)
relevant (auditory context)
irrelevant (visual context)
relevant, consistent
relevant, exploratory
irrelevant, consistent
irrelevant, exploratory
time from stimulus onset [s]
decoding
mouse 2
subspace projection
mouse 2
behaviour relevant
mouse 2
behaviour irrelevant
mouse 2
decoding
mouse 3
subspace projection
mouse 3
behaviour relevant
mouse 3
behaviour irrelevant
mouse 3
decoding
mouse 4
subspace projection
mouse 4
behaviour relevant
mouse 4
behaviour irrelevant
mouse 4

**Supplementary Figure 2**. **Stimulus decoding for individual ACC implanted mice in various task and behavioural conditions**, (n=4 mouse  in double rows). **A**, Smoothed (10 ms resolution, ma=51 points, longer than the mean on Fig. 1, for clarity of view) time course along the trial of decoding accuracy from neurons of visual (*top, blue*) and auditory (*bottom, green*) stimuli when they are relevant in their respective context (*light color*), or irrelevant in the opposite context (*dark color*). Mean (*lines*) and s.e.m. (*bands*) of cross-validation folds. **B**, similar to a *A*, but activity from go and nogo trials projected onto the DV of stimulus decoder from *A*, then absolute difference between average activity (*lines*) and the sum of s.e.m.-s (*bands*) of 'go' and 'no go' trials plotted. Note that standard deviation rather than s.e.m. determines discriminability of individual trials. **C**, Similar to *A*, but trials in the relevant context were stratified into consistent (*purple*) and exploratory (*orange*) groups according to the behavioural criteria detailed in Fig. 1C, while stimulus differentiating colors are omitted. **D**, as *C*, but in the irrelevant context.

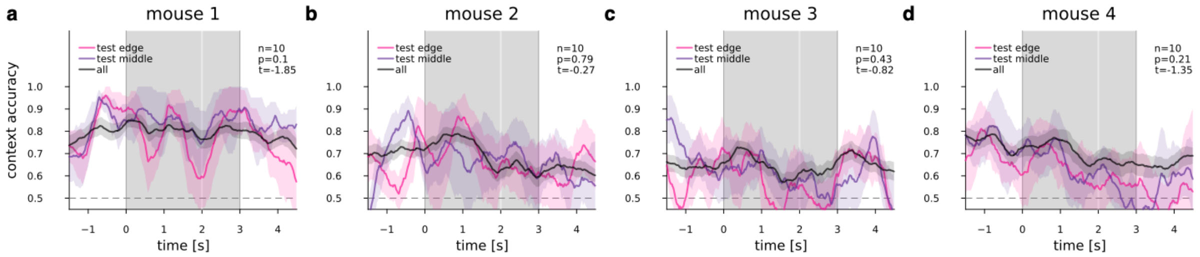


**Supplementary Figure 3**. **Context decoding is robustly invariant to session position. A-D**, Context decoder accuracies at each time point along the trial (smoothed for display purposes, ma=51) with three different cross-validation schemes, per mouse (*panels*). We trained context decoders in trials either in the middle of the entire session (end of the first context block and beginning of the second context block respectively) leaving out 10-10 trials per context at the edges (beginning and end respectively) for testing at 2 CV folds (*pink*, *line* and *band* for mean and s.e.m. over CVs) or trained at the edges and tested in the middle 10-10 trials (*purple*). Control decoder accuracies from Fig. 3A with the 10-fold CV (*black*). Block averaged paired t-test statistics over 0.6 s block width show all p>0.1 between edge test and middle test in all mice.

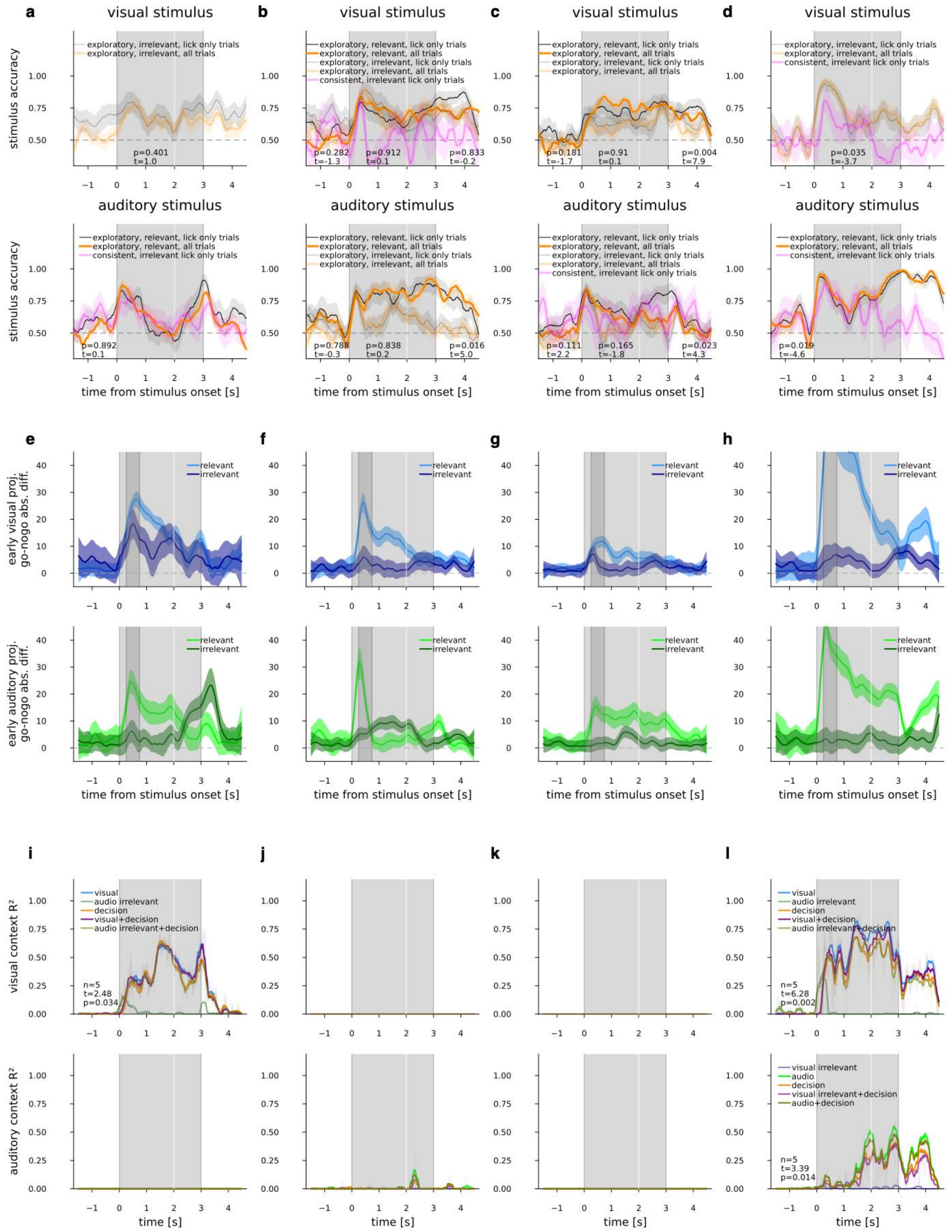


**Supplementary Figure 4**. **Controlling for licking patterns**. *Columns*: n=4 mice with ACC electrode implant. **A-D**, Effect of constraining stimulus decoders to lick-only trials. Visual (*top*) and auditory (*bottom*) stimulus decoder accuracies (mean and s.e.m. over CVs) in the irrelevant context in exploratory trials in licking-only (*black lines*) and all trials (*orange lines*, same as Supplementary Fig. 2F orange lines) in the relevant (*saturated color*) irrelevant (*faint color*) context. Lick-only consistent trials are also displayed as comparison (*purple lines*). If the number of either 'go' or 'no go' trials was below 10, the decoder was excluded from the analysis. Note that this analysis relied on error trials, as false alarms contributed to constructing the decoders. Paired t-statistics (left to right, on each panel

where applicable) are comparisons between exploratory relevant lick only vs. all, exploratory irrelevant lick only vs. all, and exploratory lick only relevant vs. irrelevant conditions respectively, using block-averaging between 0.6-3 s of 0.6 s blockwidth (n=4). Decoder accuracy time courses were smoothed (ma=31) for display purposes only. **E-H**, Projection of activity onto the stimulus-only subspace. Visual (*top, blue*) and auditory (*bottom, green*) mean (*lines*) and s.e.m. (*bands*) of absolute activity difference between successful go and nogo trials, projected onto the stimulus subspaces. Stimulus subspaces were calculated from stimulus decoder DVs between 0.25-0.75 second (*dark grey shading*) of stimulus presentation (light grey shading), where neither suppression, nor choice-related activity was present. When in a relevant context, colors are lighter. **I-L**, Explained variance of predicting the firing rates of individual neurons from either stimuli, choice or both in both contexts at each timepoint in consistent trials. Best successfully predicted neurons (neurons with $R^2 > 0$, mean over cross-validation folds) at each timepoint (*lines*). Visual context (*top*) and auditory context (*bottom*). The p values correspond to the difference between choice + relevant stimulus predictor (*purple* in visual context, *olive* in auditory context) from choice + irrelevant stimulus predictor (*olive* in visual context, *purple* in auditory context) over time blocks (n=4, block-averaging 0.6s windows between 0.6-3s) where the mean contained at least one predictable neuron in both predictions throughout stimulus presentation (one-sampled one-tailed t-test, mean differences were positive in all blocks). Timepoints throughout lines where none of the neurons were predictable were linearly interpolated for display purposes, but not taken into account for statistics. Mice and context blocks where no neurons were predictable for at least one block were omitted from the statistics in panels (3 predictable out of 8).
